# Spontaneous intraperitoneal rupture of hepatic hydatid cyst: a rare cause of ascites

**DOI:** 10.1186/1471-2482-14-99

**Published:** 2014-11-26

**Authors:** Manel Limeme, Sana Yahyaoui, Houneida Zaghouani, Mosaab Ghannouchi, Abdelmajid Khnissi, Habib Amara, Rached Letaief, Dejla Bakir, Chakib Kraiem

**Affiliations:** Department of Radiology, Farhat Hached hospital, 4000 Sousse, Tunisia; Department of General Surgery, Farhat Hached hospital, 4000 Sousse, Tunisia

**Keywords:** Hydatid disease, Spontaneous rupture, Liver

## Abstract

**Background:**

Hydatid disease is endemic in certain areas of the world and it is located mostly in the liver. Intraperitoneal rupture is rare. Rupture may result from trauma or may occur spontaneously from increased pressure of the cystic fluid. Ruptured hydatid cyst is a rare cause of ascites, but should be considered in the differential diagnosis, especially in endemic areas. The diagnosis of ruptured hydatid cyst should be prompt because it requires emergency intervention.

**Case presentation:**

The present case refers to a 62 year old Tunisian male admitted in our institution for diffuse abdominal distension. Physical examination was unremarkable except for the presence of ascites. Abdominal ultrasonography showed a large amount of fluid into the peritoneal cavity associated with many intraperitoneal cysts with a scalloping on the liver. It showed also a heterogeneous cystic lesion of the segment II of the liver. Abdominal computed tomography (CT) revealed in addition a fat infiltration and a thickening of the peritoneum. Thus intraperitoneal hydatid cyst rupture was suspected and emergency laparotomy was performed. A yellow serous fluid , containing many daughter vesicles disseminated through the peritoneal cavity was noted. A mass consistent with a hydatid cyst was noted at segment II of the liver with a tear on the inferior surface. Thus, intraperitoneal rupture of hepatic hydatid cyst was diagnosed.

**Conclusion:**

The rupture of hydatid cyst into the peritoneal cavity is rare but presents a challenge for the radiologist and the surgeon. This condition is included in the differential diagnosis of ascites in endemic areas.

## Background

Human hydatid disease usually occurs by infestation with Echinococcus granulosus. Humans are affected by enteral dissemination and become accidental intermediate hosts [1]. Hydatid disease is an endemic problem in Tunisia as well as in sheep-bearing regions in the world. It may be located in any organ of the body. The most frequently involved organ is the liver (50% to 70%) [2]. Complications occur in 5 to 40% patients with hepatic cysts and The reported frequency of hydatid cyst of the liver ruptured into the peritoneal cavity ranges from 1 to 16% [3]. Ascites is rarely seen in hydatid disease. In this study, we present a rare case of spontaneous rupture of hepatic hydatid cyst into the peritoneum, in which the patient was admitted for a massive ascites.

## Case presentation

**Figure 1:**
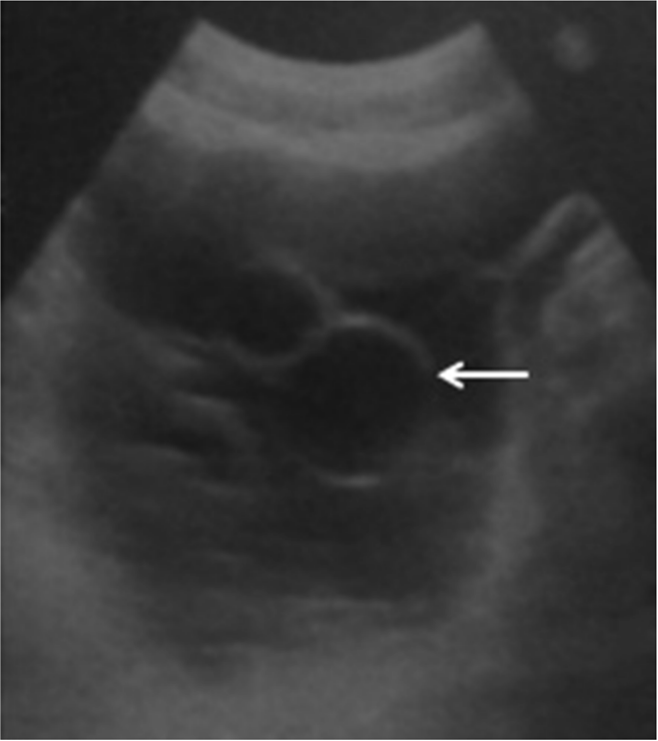
**Abdominal ultrasonography shows a large amount of fluid and cysts into the peritoneal cavity (Arrow).**

**Figure 2:**
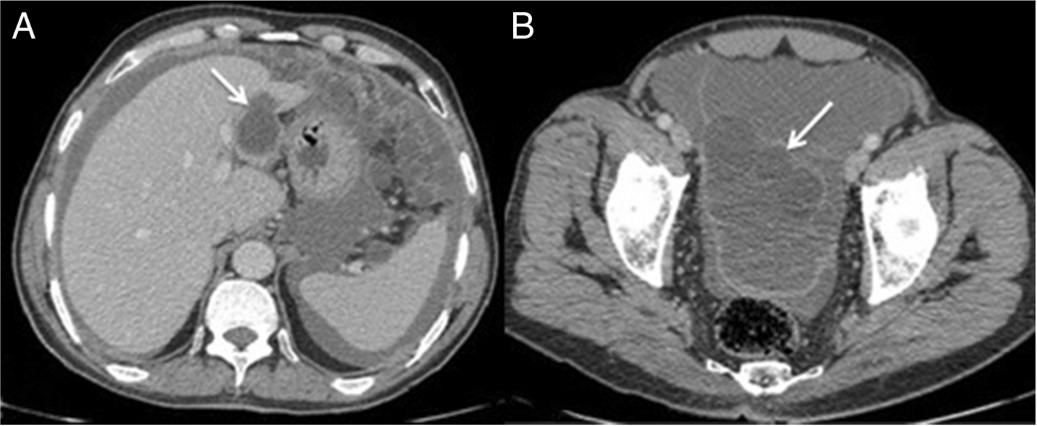
**Axial contrast-enhanced CT scan. A**. Well-defined and thin walled cystic lesion in the segment II of the liver (Arrow). **B**. Widespread ascites and cysts into the peritoneal cavity (Arrow).

**Figure 3:**
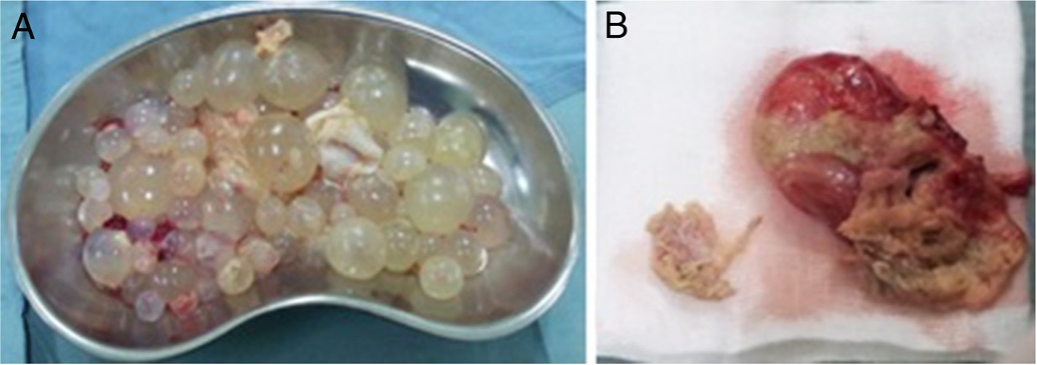
**Macroscopical findings of the specimen.** View of multiple cysts removed from peritoneum **(A)** and the ruptured liver cyst **(B)**.

A 62 year old Tunisian male was hospitalized for exploration of abdominal distension in the preceding two months with progressive aggravation. The patient had no history of trauma, surgery or systemic disease earlier. The patient had no history of trauma, prior surgery or systemic disease. Physical examination found ascites without any other abnormality. The ascitic fluid was thick and yellow with high level of lipids. Because the ascites was so massive and chylous, without any sign of hepatic, cardiac or nephrological disorder, tuberculosis or neoplasic causes were suspected. Abdominal ultrasonography showed a large amount of fluid into the peritoneal cavity with associated with many intraperitoneal cysts with a scalloping on the liver (Figure 1). It showed also a heterogeneous cystic lesion in the segment II of the liver measuring 3.5 × 2.5 cm. The biliary ducts have a normal calibre. Abdominal computed tomography (CT) before and after intravenous administration of contrast agent revealed in addition a fat infiltration and a thickening of the peritoneum (Figure 2). Thus an intraperitoneal rupture of a hydatid cyst was suspected and an emergency laparotomy was performed. Yellow serous fluid (2000 ml) was noted, containing many daughter vesicles disseminated through the peritoneal cavity. A mass consistent with a hydatid cyst was noted at segment II of the liver with a tear on the inferior surface (Figure 3). After a partial cystectomy, the cyst pouch was irrigated with hypertonic saline (3%), and the peritoneal cavity was washed with isotonic saline for 10 to 15 minutes. After surgery, the patient was started on albendazole (10 mg/kg) for three months.

## Discussion

Echinococcus granulosus is endemic in many sheep- and cattle-raising countries throughout the world. Definitive hosts are the canines (usually dogs). Intermediate hosts are usually grazing animals (sheep or cattle) or humans. Humans may become intermediate hosts through contact with a definitive host or the ingestion of contaminated water or vegetables. The usual locations are the liver and the lung. Other sites including the peritoneum are uncommon [4].

Rupture of hydatid cyst of the liver is uncommon and may result from trauma or may occur spontaneously from increased pressure of the cystic fluid [5]. Our patient had no history of trauma before admission. This rare condition occurs most often in younger patients, specifically those with superficially located and bigger lesions. When both the pericyst and the endocyst are torn, cyst contents escapes by a nonanatomic route into the peritoneal space: it’s called a direct rupture; and hydatid fluid, brood capsules and scolices are spilled into the peritoneal cavity. A long-term sequela of direct rupture into the peritoneal cavity is implantation of scoleces leading to metastatic hydatidosis [6].

The rupture of a hydatid cyst in the peritonium has two clinical forms with distinct outcomes: the minimal fissuring and the massive ruptures. The former, is the more frequent, and it is caused by a trauma which is often unrecognized and neglected.

The usual clinical presentation is a progressive increase of the abdomen volume associated or not with a transient cutaneous eruption. Because the hydatid liquid is in feeble quantity in the great peritoneal cavity, it can either encysts and give rise to an encysted vesicular peritoneal echinococcus, or stay free resulting in a real military hydatid disease. The massive forms are much rarer and are found after a great effort. They make result in a rapid and complete draining of the cyst in the peritoneal cavity. They can either evolute at low rate realizing a subacute form or in a rapid way realizing an acute form including abdominal pain, urticaria, anaphylaxis and sudden death [6, 7].

The diagnosis of ruptured hydatid cyst should be prompt because it requires an emergency intervention [5]. Preoperative diagnosis requires a complex work-out including ultrasound scan, CT, and serological tests [8].

Ultrasound is a non invasive method. It is useful for identifying intra-abdominal fluid and a cyst with detached membrane [5, 9]. CT is the modality of choice to diagnosis; because it allows imaging of the entire abdomen and pelvis. The direct rupture of the hepatic hydatid cyst into the peritoneal cavity can be detected on CT by various findings such as detached membranes, reduction of the cyst size, cyst wall discontinuity and change in the architecture of the hydatid cyst. These data are the imaging's features of ruptured hydatid cyst [10]. The presence of intra-abdominal fluid and the presence of daughter cysts into the peritoneal cavity can also be diagnosed by CT [11]. In the present case, typical ultrasound and CT findings were found.

Serology is used to detect specific serum antibodies or circulating antigens by a variety of immunodiagnostic methods. The most commonly used technique is enzyme linked immunosorbent assay (ELISA) for detection of echinococcal antibodies (IgG) in the serum. A false positive value can occur in a normal pe-son especially in endemic area and similar results can also occur in patients with other parasitic infestations [12].

The perforation of the hydatid cyst may cause dissemination of the parasite and increased morbidity and mortality rate. Immediate medical treatment against allergic reactions should be initiated, and emergency surgery should be performed after diagnosing rup-ture of hydatid cysts [2].

The goal of the surgical treatment is to prevent complications, to eliminate local disease, and to minimize morbidity, mortality, and recurrence rates. Surgery is still the main modality for the treatment of hydatid disease, despite the developments in radiologic techniques and medical therapy. However, controversy exists about the choice of a radical versus a conservative approach; radical operations include pericystectomy and liver resection, whereas conservative techniques include external drainage, unroofing, and cavity-obliterating methods. Generally, conservative methods are favored in endemic areas [2].

All of the cyst contents should be removed; the cyst and peritoneal cavity should be washed with saline solution and scolicidal agents. Hypertonic saline (3%, 15%, or 30%) is scolocidal [5].

To prevent recurrences, medical treatment is mandatory. It should be started immediately after surgery and continued for 1 to 6 months, depending on individual circumstances, to reduce the risk of recurrence. Albendazole (10–15 mg/kg/d) is the drug used most commonly in previous studies and was used in the present patient [1, 12].

A ruptured hydatid cyst requires a meticulous postoperative follow-up. Patients may be followed with ultrasonographic examination and indirect hemagglutination test starting 6 months after surgery and repeated every 1 to 2 years. A CT scan may show a recurrence. Cysts that were overlooked during surgery may be erroneously interpreted as recurrences during long-term follow-up. Recurrences may also occur because of insufficient surgery or medical treatment after rupture of a hydatid cyst [3].

## Conclusions

The rupture of a hydatid cyst into the peritoneal cavity is rare but presents a challenge for the radiologist and the surgeon. This condition is included in the differential diagnosis of ascites in endemic areas. Specific imaging findings on ultrasonographic examination and CT allow definite diagnosis. Emergency surgery is the main treatment for intraperitoneal rupture of hydatid cysts, and medical treatment should be given postoperatively.

## Consent

Written informed consent was obtained from the patient for publication of this case report and any accompanying images. A copy of the written consent is available for review by the Editor of this journal.

## Abbreviations

CT: Computed tomography
ELISA: Enzyme linked immunosorbent assay
IgG: Immunoglobulin G.
